# On the Characters and Structural Peculiarities of a Group of Morbid Growths in Which Cancerous Affections Are Included

**Published:** 1843-11

**Authors:** 


*On the Characters and Structural peculiarities of a Group of Morbid Growths in which Cancerous Affections are included.* By Dr. Hodgkin.*—This paper was in continuation of a subject which had already been brought before the society on a former occasion by the same writer.

After describing the different appearances revealed by the improved microscopes of the present day, the author endeavoured to connect the nucleated cells, which Muller has shown to exist in these structures, with the production of those compound cysts which were described in the former paper, and pointed out as affording the type of the adventitious structure referred to.

The following are the conclusions which the author is desirous of drawing from the observations contained in the paper:

1st. The unrestricted confirmation of the views contained in the former paper as to the existence of the type of compound serous cysts in the adventitious structures referred to. The author had not only found it in man, but in several of the inferior species of mammalia, and in birds. Several able observers had, on examination, coincided with his views, and he mentioned the late Professor Delpech, and the present Professor Rokitanski, having personally informed him of their having, independently, been induced to adopt his views.

2d. That the microscopic examination of these tissues, though extremely interesting, does not furnish perfectly conclusive tests of any particular form of adventitious structure to which a specimen may belong, but that it demonstrates the application of the nucleated cell theory, whilst it is fatal to that of cancerous matter being formed in the blood and

* Meetings of Royal, Medical, and Chirurgical Society.

eliminated at the spots at which the tumours become manifest. It therefore furnishes an important argument in favor of operation, though other practical considerations require to be attended to before operation is decided on.

3d. That to have a complete view of the mode of production of these structures, we must combine the cell theory of Schwann and Muller, the coagulation principle which the author had previously suggested, and the process of organization investigated by Mr. Kiernan—three stages of development which appear to occur in the order just enumerated; and that none of the phenomena, taken singly, is an adequate test of malignancy, which, as stated in his first paper, must be regarded as the sum of several characters.

4th. That chemical analysis, though extremely important and interesting, affords an imperfect and inadequate criterion, as the principles concerned may vary or be changed in the progress of development.

5th. That, in operating for the removal of a tumour of this class, it is extremely important to leave behind none of those minute cysts which often form granules in the surrounding cellular membrane, though it may appear in other respects perfectly healthy. This appears to be a mode of extension of the disease independent of inflammation.

6th. That experience teaches us that the infiltrated form of these diseases occurs in the structures in the neighborhood of the purely adventitious growth, when these structures have been the seat of inflammation, and that the chances of success from operation are consequently infinitely diminished when such surrounding inflammation has taken place. The presence of the peculiar matter of the disease in the interior of the vessels appears to be one of the modes in which infiltration, the result of inflammation, exhibits itself, and is, therefore, not a valid argument in favor of the pre-existence of such matter in the circulating blood.—*Bulletin Medical Sciences.*

